# Brain-Region-Specific Differences in Protein Citrullination/Deimination in a Pre-Motor Parkinson’s Disease Rat Model

**DOI:** 10.3390/ijms252011168

**Published:** 2024-10-17

**Authors:** Audrey Mercer, Marco Sancandi, Amy Maclatchy, Sigrun Lange

**Affiliations:** 1 Department of Pharmacology, UCL School of Pharmacy, London WC1N 1AX, UK; a.mercer@ucl.ac.uk (A.M.); marco.sancandi.16@ucl.ac.uk (M.S.); 2 Pathobiology and Extracellular Vesicles Research Group, School of Life Sciences, University of Westminster, London W1W 6XH, UK; a.maclatchy@westminster.ac.uk

**Keywords:** peptidylarginine deiminases (PADs), citrullination/deimination, Parkinson’s disease, brain, cortex, hippocampus, midbrain, striatum, cerebellum, olfactory bulb, KEGG

## Abstract

The detection of early molecular mechanisms and potential biomarkers in Parkinson’s disease (PD) remains a challenge. Recent research has pointed to novel roles for post-translational citrullination/deimination caused by peptidylarginine deiminases (PADs), a family of calcium-activated enzymes, in the early stages of the disease. The current study assessed brain-region-specific citrullinated protein targets and their associated protein–protein interaction networks alongside PAD isozymes in the 6-hydroxydopamine (6-OHDA) induced rat model of pre-motor PD. Six brain regions (cortex, hippocampus, striatum, midbrain, cerebellum and olfactory bulb) were compared between controls/shams and the pre-motor PD model. For all brain regions, there was a significant difference in citrullinated protein IDs between the PD model and the controls. Citrullinated protein hits were most abundant in cortex and hippocampus, followed by cerebellum, midbrain, olfactory bulb and striatum. Citrullinome-associated pathway enrichment analysis showed correspondingly considerable differences between the six brain regions; some were overlapping for controls and PD, some were identified for the PD model only, and some were identified in control brains only. The KEGG (Kyoto Encyclopedia of Genes and Genomes) pathways identified in PD brains only were associated with neurological, metabolic, immune and hormonal functions and included the following: “Axon guidance”; “Spinocerebellar ataxia”; “Hippo signalling pathway”; “NOD-like receptor signalling pathway”; “Phosphatidylinositol signalling system”; “Rap1 signalling pathway”; “Platelet activation”; “Yersinia infection”; “Fc gamma R-mediated phagocytosis”; “Human cytomegalovirus infection”; “Inositol phosphate metabolism”; “Thyroid hormone signalling pathway”; “Progesterone-mediated oocyte maturation”; “Oocyte meiosis”; and “Choline metabolism in cancer”. Some brain-region-specific differences were furthermore observed for the five PAD isozymes (PADs 1, 2, 3, 4 and 6), with most changes in PAD 2, 3 and 4 when comparing control and PD brain regions. Our findings indicate that PAD-mediated protein citrullination plays roles in metabolic, immune, cell signalling and neurodegenerative disease-related pathways across brain regions in early pre-motor stages of PD, highlighting PADs as targets for future therapeutic avenues.

## 1. Introduction

Molecular pathways in early stages of Parkinson’s disease (PD), including pre-motor PD, are an important area of research in the light of a lack of current treatment strategies and the need for the identification of early biomarkers [1]. More than 10 million people worldwide live with PD, the second most common age-related neurodegenerative disorder and 4% of affected people are diagnosed before the age of 50. The identification of early PD-related pathways that can serve as novel drug targets and aid early diagnosis is, therefore, of pivotal importance. In combination with clinical and post-mortem human patient samples, which are in limited supply, the use of early-stage PD animal models offers promising avenues for in vivo assessment of novel molecular pathways and candidate pharmacological lead compounds for therapeutic intervention.

Recent research has highlighted a role for post-translational citrullination/deimination of proteins caused by peptidylarginine deiminases (PADs) through the irreversible conversion of arginine to citrulline in a Ca^2+^-catalysed manner. The conversion of each arginine to citrulline leads to the loss of one positive charge and mass decrease of 1 Da, which can alter intra- and/or intermolecular protein interactions, modify protein structure and function, or cause protein denaturation depending on the extent of citrullination. Citrullination of proteins is associated with inflammatory and neurodegenerative diseases, including through altered self-epitopes, changes in protein interactions and epigenetic regulation [2]. The elevation of Ca^2+^ in many pathological processes is well documented and importantly associated with PAD-mediated citrullination in many diseases and has most recently also been linked to PD [3,4,5].

PADs are found as five isozymes in mammals, in which they show tissue-specific expression and vary in preference for target proteins, with some shared targets [6]. Furthermore, beta sheets and intrinsically disordered proteins are more susceptible to citrullination/deimination, and the position of the arginine also plays a role. Post-translational citrullination also contributes to neo-epitope formation and can increase the antigenic diversity of proteins via changes in primary, secondary and tertiary protein structures. This may lead to altered antigen processing, presentation and immune recognition, which can affect downstream signalling pathways [7,8]. Importantly, a wide range of cytosolic, cytoskeletal, mitochondrial and nuclear-associated proteins can undergo citrullination, leading to their modified functions, including loss of function, newly acquired functions and protein moonlighting. PAD2 is considered the most ancestral and ubiquitously expressed PAD isozyme, while all isozymes have been linked to physiological and pathobiological processes [6,8,9,10,11]. The main PAD isozymes studied to date in the brain and in neurodegenerative disorders have been PAD2 and PAD4, including in development and ageing [12], Alzheimer’s disease (AD) [13,14], amyotrophic lateral sclerosis (ALS), prion diseases and in multiple sclerosis (MS) [15,16,17,18,19,20,21], as well as in acute brain injury [22,23,24,25]. However, importantly, PADs have also recently been highlighted in human PD brain samples, iPSC models and in PD animal models [4,5,26]. Roles for PAD3 have been described in CNS regeneration [27], in brain cancer [28,29], in neuronal stem cells [30] and in neuronal viral infection [31]. Although the roles of PAD3 in neurodegeneration remain unclear, recent research highlighted elevated PAD3 levels in PD post-mortem human brain samples [5] as well as in pre-motor PD rat cortex and hippocampus [4]. PAD1 has been associated with skin diseases, development and some cancers [32,33,34,35]. Importantly, PAD1 was also recently identified by our group to be elevated in human post-mortem PD brain samples of the hippocampus and cortex [5]. PAD6 is shorter than the other isozymes, shares less sequence homology with the other PAD family members and does not seem to bind calcium, contrary to the other isozymes [36]. PAD6 has mainly been studied for roles in early embryonic development, oocyte formation and embryo implantation [37,38], and some associations have been made with cancers [39]. Recently, possible roles for PAD6 in the CNS have been highlighted, including in brains of hypoxia-challenged naked mole-rats [40] and in post-mortem human PD brain samples where PAD6 was detected in the brain vasculature by immunohistochemistry [5].

As recent studies from both animal models and human post-mortem brain samples have identified increased citrullination and differences in PADs at early PD stages [4,5], an investigation is now warranted into the brain-region-specific citrullinomes in early stages of PD. The current study, therefore, used the previously published 6-hydroxydopamine (6-OHDA)-induced pre-motor PD rat model [4] to assess changes in citrullinated protein targets by proteomic analysis in the following brain regions: striatum, olfactory bulb, midbrain, hippocampus, cortex and cerebellum.

## 2. Results

### 2.1. Isolation of Citrullinated/Deiminated Target Proteins from Brain Regions and Silver Staining

**Figure 1:**
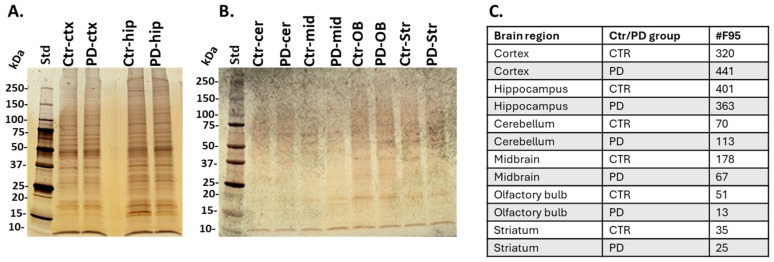
Citrullinated proteins, isolated by F95 enrichment from (**A**) cortex (ctx) and hippocampus (hip), and (**B**) cerebellum (cer), midbrain (mid), olfactory bulb (OB) and striatum (str). F95 enriched fractions are shown from sham/control (ctr) and pre-motor PD (PD) brain regions on a silver-stained SDS-PAGE TGX 4–20% gel. (**C**) A summary of numbers of citrullinated proteins (#F95) identified per brain region and with respect to the experimental group (control/sham (white)—ctr; PD-group (grey)) by LC-MS/MS analysis.

Total citrullinated/deiminated proteins were isolated from the brain tissue of the six brain regions under study using the pan-citrulline F95 antibody and the catch-and-release immunoprecipitation kit, according to previously described methods [40]. The yield of citrullinated proteins was highest in cortex and hippocampus, as observed by silver staining of eluted fractions (Figure 1A), and citrullinated proteins were isolated at lower yield from the cerebellum, midbrain, olfactory bulb, and striatum, as observed by silver staining of the F95 enriched eluted proteins from each brain region (Figure 1B). The numbers of citrullinated protein targets identified by LC-MS/MS analysis per brain region in control and PD groups are further summarised in Figure 1C. The highest numbers of citrullinated protein hits were detected in the cortex and hippocampus, followed by the cerebellum, midbrain and olfactory bulb, with the fewest targets identified in the striatum (Figure 1C). Comparing individual brain regions from the control and PD groups, elevated numbers of total citrullinated hits were seen in the PD model for the cortex and cerebellum (Figure 1C). Full lists of citrullinated target proteins identified in all six brain regions of the control and PD groups, respectively, are provided in Supplementary Tables S1–S12.

### 2.2. Identification of the Brain-Region-Specific Citrullinomes by LC-MS/MS

**Figure 2:**
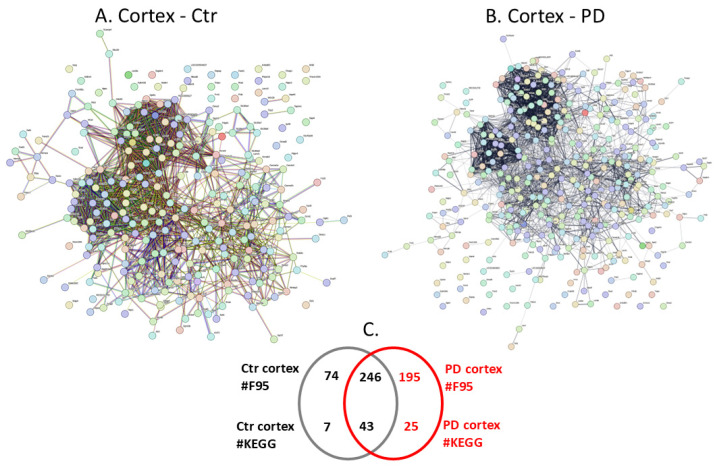
The cortex citrullinome. Citrullinated/deiminated proteins identified in the control and pre-motor PD rat cortex. (**A**) Protein interaction network of the protein citrullinome of sham/control rat cortex. (**B**) Protein interaction network of the protein citrullinome of PD rat cortex. (**C**) A Venn diagram representing numbers (#) of citrullinated protein hits (F95) and of associated KEGG pathways in sham/control and pre-motor PD cortex.

**Figure 3:**
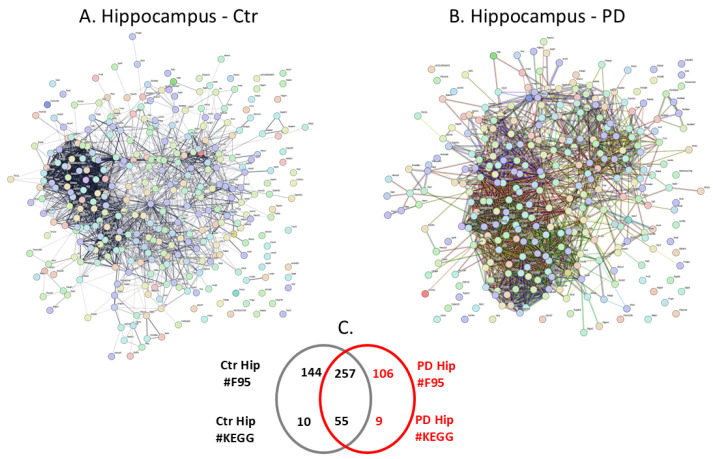
The hippocampal citrullinome. Protein–protein interaction networks of the protein citrullinome of sham/control (**A**) and PD (**B**) rat hippocampus. (**C**) The Venn diagram represents numbers (#) of citrullinated protein hits (F95) and KEGG pathways in the hippocampus.

**Figure 4:**
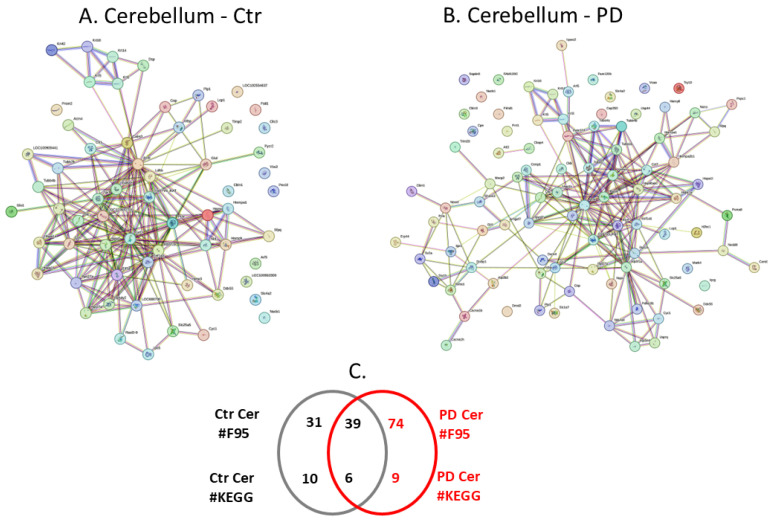
The cerebellar citrullinome. Protein interaction networks of the protein citrullinome of control (**A**) and PD (**B**) rat cerebellum. (**C**) The Venn diagram summarises numbers (#) of citrullinated protein hits (F95) and KEGG pathways.

**Figure 5:**
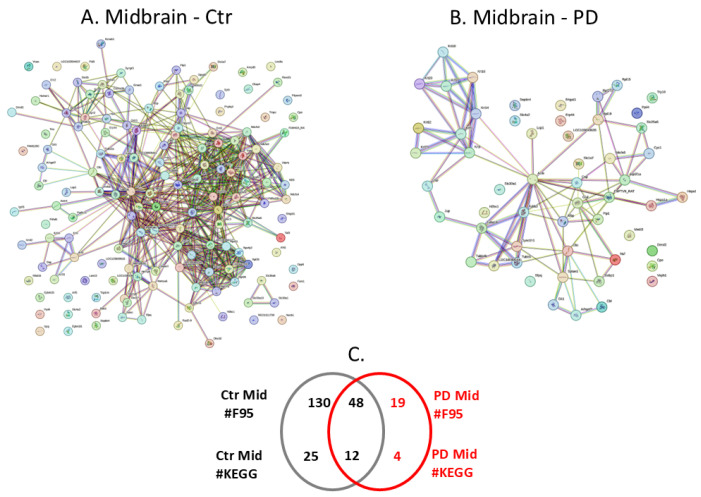
The midbrain citrullinome. Protein interaction networks are shown for the control (**A**) and PD (**B**) rat midbrain. (**C**) Numbers (#) of citrullinated protein hits (F95) and associated KEGG pathways are presented in the Venn diagram.

**Figure 6:**
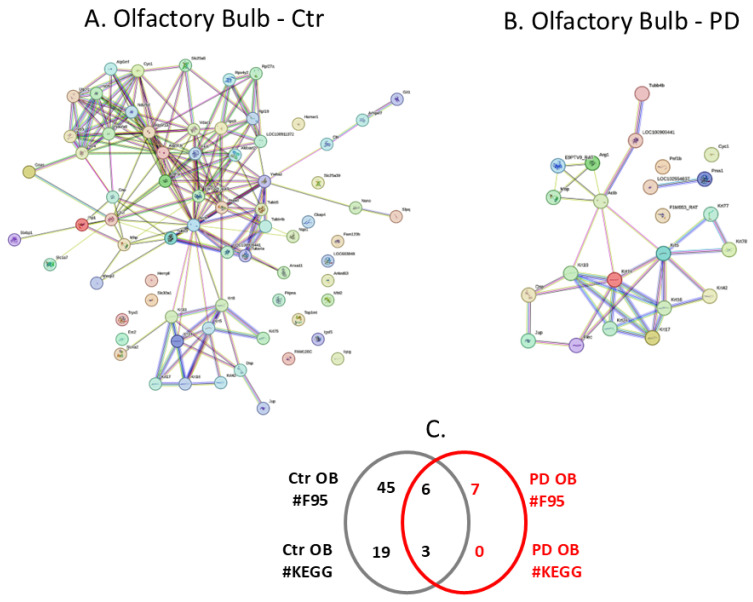
The olfactory bulb citrullinome. (**A**) Protein interaction networks of the protein citrullinome of control rat olfactory bulb. (**B**) Protein interaction network of the protein citrullinome of PD rat olfactory bulb. (**C**) The Venn diagram represents the numbers (#) of citrullinated protein hits (F95) and KEGG pathways associated with the control and pre-motor PD olfactory bulb citrullinomes, respectively.

**Figure 7:**
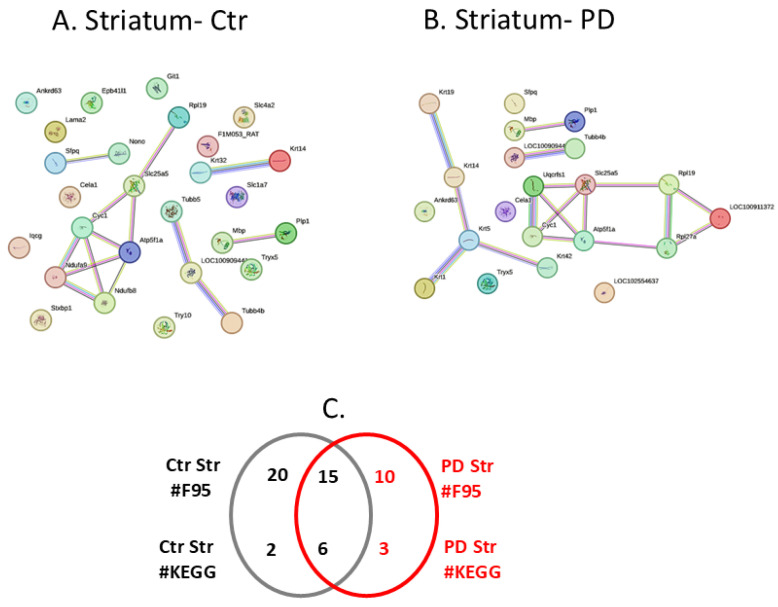
The striatum citrullinome. Citrullinated/deiminated proteins identified in sham/control and pre-motor PD rat striatum. (**A**) Protein interaction networks of the protein citrullinome of control rat striatum. (**B**) Protein interaction network of the protein citrullinome of PD rat striatum. (**C**) A Venn diagram representing citrullinated protein hits (F95) and the number of associated KEGG pathways in control and pre-motor PD striatum.

Citrullinated proteins isolated per brain region were further assessed by LC-MS/MS analysis for the identification of protein target hits. Individual and shared protein hits are summarised for the different brain regions in the Venn diagrams in Figure 2C, Figure 3C, Figure 4C, Figure 5C, Figure 6C and Figure 7C, which represent the cortex (Figure 2), hippocampus (Figure 3), cerebellum (Figure 4), midbrain (Figure 5), olfactory bulb (Figure 6) and striatum (Figure 7). Further details on the individual protein hits per brain region (control and PD model, respectively) are provided in Supplementary Tables S1–S12. Citrullinated protein hits were then used to create protein–protein interaction networks for the brain-region-specific “citrullinomes”, representative of all citrullinated/deiminated proteins identified in each brain region, using STRING analysis (https://string-db.org/; accessed on 18–19 March 2024). The resulting protein–protein interaction networks are shown in Figure 2, Figure 3, Figure 4, Figure 5, Figure 6 and Figure 7 for the cortex (Figure 2), hippocampus (Figure 3), cerebellum (Figure 4), midbrain (Figure 5), olfactory bulb (Figure 6) and striatum (Figure 7). All KEGG pathways identified in association with the brain-region-specific citrullinomes for the six brain regions, as shown in Figure 2, Figure 3, Figure 4, Figure 5, Figure 6 and Figure 7 (and presented in Section 2.3.1, Section 2.3.2, Section 2.3.3, Section 2.3.4, Section 2.3.5 and Section 2.3.6), are summarised in Table 1.

### 2.3. Protein–Protein Interaction Network Analysis for the Brain Region-Specific Citrullinomes in Pre-Motor PD versus Control Brains

The protein–protein interaction networks for the citrullinated proteins identified in each brain region were created in STRING (Figure 2, Figure 3, Figure 4, Figure 5, Figure 6 and Figure 7), comparing the sham/control and pre-motor PD groups for all six brain regions under study (Figure 2 cortex; Figure 3 hippocampus, Figure 4 cerebellum, Figure 5 midbrain, Figure 6, olfactory bulb and Figure 7 striatum). The extent of the protein–protein interaction networks and associated pathway enrichment analysis reflected the differences in the citrullinated protein hits identified in the six brain regions. Overall, the cortex had the highest number of citrullinated protein hits, followed by the hippocampus, cerebellum, midbrain, olfactory bulb and striatum. 

#### 2.3.1. The Cortex Citrullinome and Associated KEGG Pathways

The protein citrullinome networks for the cortex, in both shams/controls and PD groups, are shown in Figure 2A,B, respectively. As summarised in the Venn diagram in Figure 2C, 195 protein hits were identified as citrullinated targets specific to the PD cortex, while 74 were only identified in the control cortex, and 246 citrullinated protein hits were common to both the control and PD cortex. The KEGG pathways associated with the cortex citrullinome were 25 for the PD cortex and 7 for the control cortex, and a further 43 KEGG pathways were identified as common between the control and PD cortex; these are listed in Table 1. The 25 KEGG pathways, which were identified as associated with the citrullinome of the pre-motor PD cortex but not found associated with the control cortex citrullinome, were the following metabolic pathways: “Inositol phosphate metabolism”; “2-Oxocarboxylic acid metabolism”; “Nitrogen metabolism”; “Pentose phosphate pathway”; “Fructose and mannose metabolism”; “Alanine, aspartate and glutamate metabolism”; “Cysteine and methionine metabolism”; “Arginine biosynthesis”; “Vasopressin-regulated water reabsorption”; “Gastric acid secretion”; “Phosphatidylinositol signalling system”. The pathways relating to cell adhesion, cell survival and cellular communication were as follows: “Rap1 signalling pathway”; “SNARE interactions in vesicular transport”; “Tight junction”; “Regulation of actin cytoskeleton”. Immune-related pathways were: “NOD-like receptor signalling pathway”; “Leukocyte transendothelial migration”; “Viral carcinogenesis”; “Platelet activation”; “Yersinia infection”; “Human cytomegalovirus infection”. The developmental pathways were as follows: “Progesterone-mediated oocyte maturation”; “Oocyte meiosis”. The neuronal and neurodegenerative associated pathways were as follows: “Axon guidance”; “Spinocerebellar ataxia”.

#### 2.3.2. The Hippocampal Citrullinome and Associated KEGG Pathways

The protein citrullinome networks for the hippocampus from the sham/control and PD groups are shown in Figure 3A,B, respectively. As summarised in the Venn diagram in Figure 3C, 106 protein hits were identified as citrullinated targets specific to the PD hippocampus, while 144 were only identified in the control cortex, and 257 citrullinated protein hits were common to both the control and PD hippocampus. The KEGG pathways associated with the hippocampus citrullinomes were 9 for the PD hippocampus and 10 for the control hippocampus, and a further 55 KEGG pathways were identified as common between the control and PD hippocampus; these are listed in Table 1. The 9 KEGG pathways identified in the PD hippocampus, but not in the citrullinome of the sham/control hippocampus, were the following metabolic pathways: “Phosphatidylinositol signalling system”; “Endocrine and other factor-regulated calcium reabsorption”; “Choline metabolism in cancer”; “Thyroid hormone signalling pathway”. The pathways relating to cell growth, development and stem cell regulation were as follows: “cGMP-PKG signalling pathway”; “Hippo signalling pathway”; “Oocyte meiosis”. The immune-related pathways were as follows: “Fc gamma R-mediated phagocytosis”. The CNS-related pathway was “Long-term depression”.

#### 2.3.3. The Cerebellar Citrullinome and Associated KEGG Pathways

The protein citrullinome networks for the cerebellum from the sham/control and PD groups are shown in Figure 4A,B, respectively. As summarised in the Venn diagram in Figure 4C, 74 protein hits were identified as citrullinated targets specific to the PD cerebellum, while 31 were identified to be specific to the control cerebellum, and 39 citrullinated protein hits were common to both the control and PD cerebellum. The KEGG pathways associated with the cerebellum citrullinomes were nine for PD, ten for control, and a further six KEGG pathways were identified as common between the control and PD cerebellum; these are all listed in Table 1. The nine KEGG pathways identified in the PD cerebellum, but not in the citrullinome of the control cerebellum, were the following metabolic pathways: “Oxidative phosphorylation”; “Thermogenesis”. The CNS and neurodegenerative-linked pathways were as follows: “Synaptic vesicle cycle”; “Alzheimer’s disease”; “Glutamatergic synapse”; “Gap junction”. The inflammatory pathways were as follows: “Endocytosis”; “Phagosome”; “Apoptosis”.

#### 2.3.4. The Midbrain Citrullinome and Associated KEGG Pathways

In the midbrain, fewer citrullinated protein hits were identified in the PD model than in the controls/shams. The protein citrullinome networks for midbrains of control and PD groups are shown in Figure 5A,B, respectively. As summarised in the Venn diagram in Figure 5C, 19 protein hits were identified as citrullinated targets specific to the PD midbrain, while 130 were only identified in the control midbrain, and further 48 citrullinated protein hits were common to both the control and PD midbrain. The KEGG pathways associated with the midbrain citrullinomes were 4 for PD only, 25 for control, and a further 12 KEGG pathways were identified as common between control and PD midbrain; these are listed in Table 1. The four KEGG pathways identified to be specific for the citrullinome of PD midbrain but not associated with the citrullinome of the control midbrain were inflammatory related: “Apoptosis”; “*Staphylococcus aureus* infection”; “Bacterial invasion of epithelial cells”. In addition, “Arrhythmogenic right ventricular cardiomyopathy” was unique to the PD model.

#### 2.3.5. The Olfactory Bulb Citrullinome and Associated KEGG Pathways

The protein citrullinome networks for olfactory bulbs from the sham/control and PD groups are shown in Figure 6A,B, respectively. As summarised in the Venn diagram in Figure 6C, 7 protein hits were identified as citrullinated targets specific to the PD olfactory bulb, while 45 were only identified in the control olfactory bulb, and a further 6 citrullinated protein hits were common to both the control and PD olfactory bulb. The KEGG pathways associated with the olfactory bulb citrullinomes were the three shared between the control and PD model, while none were specific to the PD model only, and an additional 19 citrullinome-associated pathways were identified for the control olfactory bulb; these are listed in Table 1.

#### 2.3.6. The Striatum Citrullinome and Associated KEGG Pathways

The protein citrullinome networks for the striatum from the sham/control and PD groups are shown in Figure 7A,B, respectively. As summarised in the Venn diagram in Figure 7C, 10 protein hits were identified as citrullinated targets specific to the PD striatum, while 20 were only identified in the control striatum, and a further 15 citrullinated protein hits were common to both the control and PD striatum. The KEGG pathways associated with the striatum citrullinomes were three for PD only, two for control, and a further six KEGG pathways were identified as common between the control and PD striatum; these are listed in Table 1. The three KEGG pathways identified in PD striatum, but not in the citrullinome of the control striatum, were as follows: “Ribosome”; “Oestrogen signalling pathway”; “*Staphylococcus aureus* infection”.

#### 2.3.7. Pathway Enrichment Analysis of the Brain-Region-Specific Citrullinomes Comparing Control/Sham and PD Model

**Figure 8:**
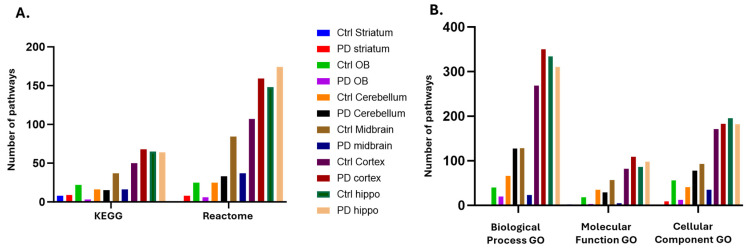
Pathway enrichment analysis of the brain-region-specific citrullinomes of control and pre-motor PD rat brains. (**A**) KEGG and Reactome pathways associated with the protein citrullinomes of the six brain regions assessed in control and PD groups. (**B**) Biological GO, Molecular Function GO and Cellular Component GO pathways associated with the protein citrullinomes of the six brain regions were assessed in control and PD groups.

In addition to the KEGG analysis, brain-region-specific citrullinomes were also assessed for Reactome pathways, and a comparison of total numbers of associated KEGG and Reactome pathways for all six brain regions from both the sham/control and PD groups is shown in Figure 8A. Reactome-associated pathways were increased in the protein citrullinome networks of the PD striatum, PD cerebellum, PD cortex and PD hippocampus compared with the control group. Reactome pathways associated with the brain-region-specific citrullinomes were reduced in the olfactory bulb and midbrain of the PD model, compared with controls (Figure 8A).

When assessing any changes in gene ontology (GO) pathways associated with the brain-specific citrullinomes, increased numbers of Biological Process GO pathways were associated with the PD cerebellum and cortex. Molecular Function GO pathways were increased in the PD cortex and hippocampus, and Cellular Component GO pathways were increased in the PD striatum, cerebellum, and cortex compared with controls (Figure 8B). There was a reduction in Biological Function GO pathways associated with the citrullinomes of the PD olfactory bulb, midbrain and hippocampus, compared with controls. Molecular function GO pathways were reduced in the citrullinomes of the PD olfactory bulb, cerebellum and midbrain, compared with the controls, while Cellular Component GO pathways were reduced in the citrullinomes of the PD olfactory bulb, midbrain and hippocampus, compared with the controls (Figure 8B).

### 2.4. Brain-Region-Related Differences in PAD Isozyme Detection

The six studied brain regions were also assessed by Western blotting (Figures S1–S3) for all five PAD isozymes (PAD1, 2, 3, 4 and PAD6), as summarised in Table 2. In the cortex, a significant increase in PAD2 and PAD3 was observed in the pre-motor PD model compared with the controls. In the hippocampus, no statistically significant changes were observed (Figure S1). In the cerebellum, a significant increase was observed for PAD3, PAD4 and PAD6 in the PD model compared with controls/shams. In the midbrain, no statistically significant changes were observed (Figure S2). In the PD olfactory bulb, a significant elevation for PAD3 was observed compared with the control/sham group. In the striatum, a significant reduction in all PADs was observed in the PD model compared with controls/shams (Figure S3).

## 3. Discussion

The detection of early molecular mechanisms in the onset of PD remains a challenge. Pre-motor PD patients display various non-motor symptoms, which include a decreased sense of smell, gastrointestinal problems, depression, sleep disturbances and autonomic dysfunction [1]. Toxin-induced rat models offer a valuable tool for investigating the pre-motor aspects of PD and exploring associated molecular alterations in distinct brain regions. Understanding brain-region-specific changes in early PD is of considerable interest, as there is mounting evidence for effects on other brain regions besides the substantia nigra (SN) and striatum, including from recent transcriptome profiling studies [41]. Previous research from our group pointed to roles for post-translational protein citrullination/deimination in the cortex and hippocampus of human post-mortem samples and in the 6-OHDA-induced rat model [4,5]. To assess roles for citrullination in early PD stages, the same pre-motor PD model was used in the current study, with a focus on six brain regions and the identification of all citrullinated/deiminated proteins, as our previous findings were limited to immunohistochemical detection of pan-citrullination in cortex and hippocampus [4,5]. Here, we assessed citrullinated proteins and PAD isozymes in the cortex, hippocampus, striatum, midbrain, cerebellum and the olfactory bulb of the 6-OHDA rat model. A downstream protein–protein interaction network analysis and associated functional enrichment analysis were carried out for the brain-region-specific citrullinomes to identify the associated KEGG and GO pathways.

### 3.1. Brain-Region-Specific Differences in Protein Citrullination/Deimination and Associated Functional Pathway Analysis

In the current study, the highest number of citrullinated protein hits was detected in the cortex and hippocampus, followed by the cerebellum and midbrain, while the fewest citrullinated targets were identified in the olfactory bulb and striatum. A considerable number of physiological and pathological KEGG and GO pathways was identified when assessing the brain-region-specific citrullinomes. Some pathways overlapped between the shams/controls and the PD model within each brain region; however, some were identified as common across various brain regions. In addition, some pathways were only identified in PD brains, and others only in control brains. In addition to considering citrullinome-associated pathways identified in PD brains only, citrullinated pathways identified in both control and PD groups, or in control brains only, should be taken into consideration. Our findings showed a loss of some citrullination-associated KEGG pathways in PD that were only identified in the controls, and this may indicate important roles for PADs in various physiological functions, which may be modified in PD and suggest unfavourable changes upon loss of citrullination of physiological pathways in PD. The various citrullinome-associated KEGG pathways identified as shared, in PD only or in controls only, are summarised and briefly discussed in Section 3.1.1, Section 3.1.2 and Section 3.1.3

#### 3.1.1. Citrullinome-Associated KEGG Pathways Identified as Shared in Sham/Control and PD Brains 

KEGG pathways, which were overlapping for some of the control and PD brain-region-specific citrullinomes and related directly to neurodegeneration, included the following: “Parkinson’s disease”; “Huntington’s disease”; “Prion disease”; “Alzheimer’s disease”; “Amyotrophic lateral sclerosis”. PADs and citrullination have been shown in all these neurodegenerative diseases, indicating shared roles in neuroinflammatory and neurodegenerative protein misfolding disorders [13,14,16,17]. The additional overlapping KEGG pathways associated with neurodegeneration were as follows: “Cellular senescence”, which is an age-associated risk factor for inducing neurodegenerative diseases, including PD [42]; “Retrograde endocannabinoid signalling”, which is a lipid-based neuromodulatory system with physiological and neurodegenerative associations in the CNS, including in PD, AD, HD, MS and ALS [43,44,45,46]; “Gap junction”, which plays critical roles in CNS signalling and has received considerable attention in PD [47]; “Regulation of actin cytoskeleton”, which is critical in regulating cellular morphology and function in normal physiology and is modulated in neurodegenerative diseases [48]; “cGMP-PKG signalling pathway”, which is critical for regulation of neuronal cell survival and apoptosis, including in PD [49]; “Synaptic vesicle cycle” and “SNARE interactions in vesicular transport” which are critical for the regulation of exo- and endocytosis of vesicles in neuronal communication and retrograde transport, also linked to LRRK2 in PD [50,51]; “GABAergic synapse” and “Dopaminergic synapse”, both of which are strongly involved in dopamine transmission in health and disease, and well-studied in PD models [52]; “Cholinergic synapse”, which plays key roles in CNS synaptic function and is associated with synaptic and axon degeneration and cognitive decline, including in PD [53,54]. Further shared KEGG pathways between some control and PD regions were “Serotonergic synapse”, which is associated with motor and non-motor PD symptoms and an identified risk factor for PD [55], and “Long-term depression”, which is well recognised as a clinical symptom in PD [56]. However, the underlying mechanisms are not well understood [57]. In addition, “Cocaine addiction”, “Alcoholism” and “Morphine addiction” were also identified as shared citrullinome enriched pathways, and these link to the dopaminergic system and neurodegenerative disease, including PD [58,59,60]. The “Oxytocin signalling pathway”, also identified as a shared citrullinome pathway, has multifaceted roles in brain function, neuroinflammation and various nervous system disorders, including PD [61,62]. “HIF-1 signalling” was also a shared citrullinome-associated KEGG pathway and has been implicated in PD relating to mitochondrial dysfunction, oxidative stress and protein degradation impairment [63,64]. Further pathways included “Endocrine and other factor-regulated calcium reabsorption” and “Calcium signalling pathways”, which underly PAD-activation but also catalyse many other neurodegenerative downstream pathways, including protein aggregation, mitochondrial and other organelle crosstalk pathways in PD [65,66,67,68]. In addition, “Ferroptosis”, also a shared citrullinome pathway, links to glia–neuron crosstalk and has potential multifaceted roles in PD pathology [69,70,71,72], while “Arginine biosynthesis” is associated with the gut-brain axis crosstalk, including in PD [73]. The identification of the “Ribosome” pathway in the shared citrullinome analysis highlights possible roles for PADs in local protein synthesis in the brain [74] and may also influence mitochondrial function, including in PD pathology [75], while “Thermogenesis” is related to mitochondrial function, synaptic transmission, neurodegeneration and activation of brown fat tissue in PD [76]. In addition, shared citrullinome KEGG pathways included “Glycolysis/Gluconeogenesis” and “Citrate cycle (TCA cycle)”, both of which are important for ATP production, mitochondrial and neuronal function and have been highlighted as targets in neurodegenerative diseases, including in PD [77,78,79]. Furthermore, “Glucagon signalling pathway”, “Type II diabetes mellitus” and “Insulin secretion”, all identified here as shared citrullinome pathways, have been identified to be linked target pathways for treatment in neuroinflammation, including in PD [80]. Mitochondrial dysfunction-associated links have also been made with “non-alcoholic fatty liver disease” [81].

Additional metabolic pathways with critical roles in cellular function and which were identified as shared between the control and PD brain citrullinomes were as follows: “Biosynthesis of amino acids”, “Carbon metabolism”, “Nitrogen metabolism”, “Pyruvate metabolism”, “Propanoate metabolism”; “2-Oxocarboxylic acid metabolism”; “Pentose phosphate pathway”; “Fructose and mannose metabolism”; “Alanine, aspartate and glutamate metabolism”; “Cysteine and methionine metabolism”; “Glyoxylate and dicarboxylate metabolism”; “Vasopressin-regulated water reabsorption”; “Gastric acid secretion”. Importantly, PD has been linked to various metabolic disorders, including the early stages of the disease [82,83]. “Arrhythmogenic right ventricular cardiomyopathy” and “Cardiac muscle contraction” were further associated with the shared KEGG citrullinomes, and this may be of interest as changes to cardiac function and cardiac dysfunction are observed in PD, with heart failure being the third leading cause of death in PD patients [84].

Shared control and PD brain citrullinome KEGG pathways linked to immunity and infection included the following: “Necroptosis”, “Endocytosis”, “Phagosome”; “Bacterial invasion of epithelial cells”; “Salmonella infection”; “Legionellosis”; “*Staphylococcus aureus* infection”; “Leukocyte transendothelial migration”; “Viral carcinogenesis”. Phagocytic and bactericidal activities are modulated in neurodegeneration, where infection with various pathogens, including bacteria and viruses, is a topic of interest in the risk associated with developing PD following infection [85]. This may also be of considerable interest in relation to long-term neurological outcomes from SARS-CoV-2 infection [86,87]. “Human immunodeficiency virus 1 infection (HIV-1)” was also a common shared citrullinome KEGG pathway and is associated with neurocognitive disorders (HIV-associated neurocognitive disorder (HAND)), characterised by synaptic loss and cognitive decline [88]. HIV infection is also thought to exacerbate age-related brain disorders, including PD [89]. In PD, neuroprotective roles have been identified for the “Oestrogen signalling pathway”, which was also one of the identified shared citrullinome KEGG pathways and may link to observed sex differences identified in PD [90,91]. “Systemic lupus erythematosus” was also a shared KEGG citrullinome pathway for control and PD brains and is an autoimmune multisystemic disease with complex interactions with PD and is also associated with other cerebrovascular diseases [92,93,94]. 

#### 3.1.2. Citrullinome Associated KEGG Pathways Identified in PD Brains Only

When excluding any overlap with other control brain regions, KEGG pathways only identified in the PD brain-region-associated citrullinomes were as follows: “Axon guidance”, which is critical to neuronal function, connectivity and repair throughout the lifespan and in neurodegeneration including in PD [95,96,97]; “Spinocerebellar ataxia”, which is a heterogeneous group of progressive neurodegenerative ataxic disorders [98] and identified here for the first time in relation to citrullination. In addition, PD-specific brain citrullinome pathways associated with metabolic and immune functions included “Inositol phosphate metabolism”, which has been linked to neuronal cytotoxicity in PD, including via increased mitochondrial Ca^2+^ [99]; “Thyroid hormone signalling pathway”, which has been linked to neurological disorders including in several PD models [100]; “NOD-like receptor signalling pathway”, which forms part of the inflammasome axis in several neurodegenerative diseases including PD [101,102,103]; “Phosphatidylinositol signalling system”, which has been linked to neuroinflammatory responses, including autophagy, and their regulation in neurodegeneration and PD [104,105]. Other PD-specific pathways included “Platelet activation”, which is related to synaptic plasticity and neuronal differentiation in various brain regions and is also reflective of inflammatory responses in neurodegeneration, including PD [106,107,108]. Further PD citrullinome-specific pathways identified here included the following: “Hippo signalling pathway”, which is an evolutionarily conserved signalling network with crucial roles in various biological processes including proliferation and differentiation, regeneration, development and immunity [109,110], and linked to early neurodegenerative processes, including in PD, AD, HD [111,112,113]; “Rap1 signalling pathway”, which has been identified as a molecular pathway in PD patients [114], also relating to blood markers in early PD [108]; “Fc gamma R-mediated phagocytosis”, which plays roles in brain development, is linked to neurodegenerative disease development [115] and has been identified as dysregulated in AD [116]; “Choline metabolism in cancer”, which may be relevant as choline metabolism has been linked to the gut-brain signalling axis, which is associated to neurodegenerative disease, including PD [117,118]. In addition, infection-related citrullinome KEGG pathways identified in PD brains only were the following: “Human cytomegalovirus infection”, which has been associated with numerous neurological disorders including PD, AD, HD, autism, ataxia and brain tumours [119] and identified as one of several infectious pathogens linked to the aetiology of PD [120]; “Yersinia infection”, which may be relevant in relation to age-associated changes in gut microbiota and possible increase susceptibility to PD [121]. Additional hormonal and developmental-related pathways in the PD brain citrullinomes included the following: “Progesterone-mediated oocyte maturation”, which has been linked to molecular mechanisms involved in PD via microarray analysis [122]; “Oocyte meiosis”, which may link to studies on the PD-associated LLRK2 kinase family, which has also been shown to have roles in oocyte meiosis via regulation of actin assembly and mitochondrial function [123]. The detection of PAD6 in all brain regions may possibly link to some of the developmental-associated KEGG pathways identified in the brain citrullinome, given its known roles in developmental processes [124,125].

#### 3.1.3. Citrullinome-Associated KEGG Pathways Identified in Sham/Control Brains Only

Some KEGG pathways were associated with the citrullinomes of the control brain regions only. This included the “Phospholipase D signalling pathway”, which plays important roles in normal brain function, including in the regulation of the synaptic vesicle cycle in neuronal communication, neuronal morphogenesis, cytoskeleton modulation, neural stem/progenitor cell differentiation, and is a suggested therapeutic target in brain disorders, including PD and AD [50,126,127,128,129]. In the control brain citrullinomes, the metabolic pathways identified were “Butanoate metabolism” and “Beta-Alanine metabolism”, both of which are associated with the gut-brain axis, including in PD [73]. Additional citrullinome KEGG pathways in control brains only included “GnRH secretion”, which is related to brain connectivity, including neuron maturation, synaptic transmission, cognition and olfaction, and has been identified as a therapeutic target in Down syndrome [130], also reported to stimulate histone citrullination and cytoskeletal dynamics [131]. Homeostasis-related pathways in the control brain citrullinome were “Collecting duct acid secretion”, which plays a role in acid-base homeostasis regulation [132]. The “Apelin signalling pathway” was also associated with the control brain citrullinome and is related to multifaceted cellular regulatory roles, including in the hypothalamus, neuronal function, neuroinflammation and neurodegenerative disease, with specific neuroprotective effects in PD pathogenesis [133,134,135,136]. The “Spliceosome” pathway was associated with the control brain citrullinome and is involved in the generation of circular RNAs and influences transcription, ageing, neuroinflammation, and oxidative stress, and has been suggested as a diagnostic and prognostic biomarker for neurodegenerative disease, including in PD [137,138]. Immune-related KEGG pathways associated with the control brain citrullinome included the “mTOR signalling pathway”, which is critical for the regulation of autophagy, apoptosis and cell proliferation in gut-brain axis signalling and plays important roles for neuronal survival and has been identified as a therapeutic target in PD [139,140,141,142]. Other immune-related pathways included “Antigen processing and presentation”, which may link to brain homeostasis but also inflammatory responses, and has recently been highlighted in autoimmune features in neurodegeneration, including in PD [143,144,145], as well as “Influenza A”, which can infect the CNS and spread through the brain, and is suggested as a possible factor inducing Lewy bodies in PD [146]. The control brain citrullinome was also associated with the “Protein processing in endoplasmic reticulum” pathway, which is critical for the biosynthesis of proteins, their folding and assembly and protein quality control, while in neurodegenerative disease, including PD, this pathway plays roles in the unfolded protein response [147,148].

Overall, our findings highlight enrichment for citrullinated proteins associated with KEGG pathways for several neurodegenerative diseases, possibly indicating shared pathogenic mechanisms which may be differently modulated by citrullination in health and disease [4,149]. It must also be considered that the extent of citrullination of the various protein targets may affect their function in different ways and play roles in several downstream pathways. Collectively, the identification of the above-listed KEGG pathways associated with citrullinated proteins in control and/or pre-motor PD brain regions highlights differences between molecular and cellular pathways modulated by this post-translational modification in the different brain regions and may be relevant to PD disease progression. 

In our previous study on the same pre-motor PD rat model [4], circulatory citrullination signatures both in plasma and plasma extracellular vesicles (EVs) and associated KEGG pathways were assessed, and some correlation to KEGG pathways identified in the different brain regions in this current study can be made. Previously we identified “Parkinson’s disease”, “Huntington’s disease”, “Prion disease”, “Alzheimer’s disease”, “Retrograde endocannabinoid signalling”, “Oxidative phosphorylation”, “Oestrogen signalling pathway”, “Non-alcoholic fatty liver disease”, “SLE”, “Complement and coagulation cascades”, “Metabolic pathways” and “Apelin signalling” as citrullinome-associated KEGG pathways in PD plasma and/or plasma-EVs [4]. Many of these KEGG pathways correspond to those identified for the citrullinomes of the PD brain regions in the current study, while some pathways overlap with the control brain samples. The identification of which pathways in the circulatory plasma citrullinome can be best correlated with relevant changes in citrullinome signatures of the brain, also with respect to specific brain regions, will need further validation and may be of considerable interest.

Studies from other groups have reported increased protein citrullination/deimination in post-mortem PD brains, including in surviving dopamine neurones in the SN, while such staining was reported not to be specifically restricted to Lewy bodies [3]. A study using F95 staining linked misfolded mutated alpha-synuclein protein to increased citrullination [150], and post-mortem analysis of prefrontal cortex from X-linked dystonia Parkinsonism patients showed increased PAD2 and PAD4 levels and histone H3 citrullination [151]. Notably, those PD-related studies did not assess all five PAD isozymes, contrary to our current study. Furthermore, an assessment of citrullinome signatures in PD brains remained to be investigated. In other neurodegenerative disease studies, PAD2 has been linked to AD, prion disease and ALS [13,14,16,17], and in these studies, other PAD isozymes have often not been assessed, partly due to previous brain studies focusing on PAD2 [12]. In the naked mole-rat, which is a hypoxia-resistant animal, all PAD isozymes and the brain citrullinome were assessed, showing increased citrullination and elevated PAD1, PAD3 and PAD6 but reduced PAD2 and PAD4, and modifications in the brain citrullinome, following hypoxia challenge [40]. Increased citrullination has also been reported in the CNS in response to blast injury and traumatic brain injury [22,152], including with respect to different citrullination levels and targets between brain regions [22]. Differences in brain citrullinome protein targets between white and grey matter have been assessed in MS versus control brains [21], and sex-related differences have been reported in AD [153].

### 3.2. PAD Isozyme Differences in Control and Pre-Motor PD Brain Regions

In the cortex, a significant increase in PAD2 and PAD3 protein levels in the PD model may be correlated with the increased citrullinated hits observed for the PD group, also reflected in increased KEGG pathways associated with the PD cortex citrullinome. These findings do align with previous immunohistochemistry staining of this region in the same model [4] and in human post-mortem PD brain samples, where, in the anterior cingulate cortex, PAD2 and PAD3 were particularly elevated at Braak stage 4 [5]. In the hippocampus, some (albeit non-significant) trends were observed for reduced PAD2, PAD3 and PAD4 levels and increased PAD6 levels in the PD group. A considerable difference was observed in the IDs of citrullinated protein hits between the PD and control hippocampus, which included 106 unique citrullinated proteins in the PD hippocampus and 144 unique ones for the control hippocampus. This was reflected in the associated differences observed in the downstream KEGG and GO pathways for the respective citrullinomes. Our findings relate somewhat to a previous immunohistochemical analysis of the hippocampus in the same model, where PAD2, 3 and 4 were detected [4]. In human post-mortem PD brain samples, we found that PAD2 and PAD3 were elevated in the PD hippocampus, particularly at Braak stage 4, while only a slight increase in PAD4 was observed. In the human PD brain samples, an increase in PAD6 was mainly linked to the brain vasculature, while PAD1 was found to be elevated in the PD hippocampus [5]. In the cerebellum, a significant increase was observed in the current study for PAD3, PAD4 and PAD6 in the PD model, indicating their possible contribution to increased citrullination hits in this brain region in the PD model and differences in the associated KEGG and GO pathway analysis of the respective cerebellum citrullinomes. In the midbrain, there were some trends for decreased PAD2 and PAD4 protein levels in the PD model, which correlates with fewer citrullinated protein hits identified in the PD midbrain, and this also correlated with fewer KEGG pathways unique to the PD midbrain, compared with controls. It may be postulated that the PAD-mediated effects on the substantia nigra (SN) at this stage may be negligible, but this will require further investigation. In a study on post-mortem human PD SN specimens, the detection of deiminated proteins in astrocytes was variable in both PD and control SN specimens; however, deiminated proteins were present in the cytoplasm of SN dopamine neurones in PD samples [3]. In the olfactory bulb, a significant increase in PAD3 was observed in the PD group, also with a trend towards increased PAD1, PAD4 and PAD6 protein levels. When assessing the citrullinomes, no specific KEGG pathways were associated with the citrullinome of the PD group only, and more citrullinated hits were overall identified in the control group, indicating that there is some modulation towards less citrullination in the olfactory bulb in the early PD model; this will require further investigation. In the striatum, a significant reduction of all PADs was observed in the PD group, which correlated with fewer identified citrullinated hits compared with the control/sham group. However, there were still some differences in the target protein and associated KEGG pathways between PD and controls, with three unique KEGG pathways associated with the PD striatum. The findings of our current study compare with our previous study, assessing cortex and hippocampal brain tissue sections from the same rat model by immunohistochemistry, where we identified the most notable increased protein citrullination by positive F95 staining in both cortex and hippocampus of the PD model, particularly in the brain vasculature, compared with controls [4]. This correlates to our citrullination enrichment results, showing the most citrullinated hits from these two brain regions. In our previous study, we had also identified by immunohistochemistry that histone H3 was particularly increased in the dentate gyrus of the rat pre-motor PD model [4], and this correlates with the findings of our current study in which LC-MS/MS analysis identified histone H3 as a citrullination target in PD hippocampus, while it was not present on the hit lists for the other brain regions. Histone H3 citrullination/deimination contributes to epigenetic regulation and extracellular trap formation (ETosis) in relation to pathogenic, autoimmune and inflammatory conditions, including brain injury [23,154]. Inhibition of pan-citrullination, including histone H3 citrullination, with the pan-PAD inhibitor Cl-amidine has been effective in reducing inflammatory responses in the CNS [23,27]. Studies from other groups have reported increased protein citrullination/deimination in post-mortem PD brains, including in surviving dopamine neurones in the SN, albeit at later stages of the disease; such staining was also reported not to be specifically restricted to Lewy bodies [3]. A study using F95 staining linked misfolded mutated alpha-synuclein protein to increased citrullination [150], and post-mortem analysis of prefrontal cortex from X-linked dystonia Parkinsonism patients showed elevation of PAD2 and PAD4 and increase in histone H3 citrullination [151]. Notably, these PD-related studies did not assess all five PAD isozymes. It must be further noted that in our previous study on the rat pre-motor PD model [4], only PAD 2, 3 and 4 were assessed by immunohistochemistry, while in this current study, all five PAD isozymes were assessed in the same rat pre-motor PD model by Western blotting. Our findings, therefore, provide some additional information on PAD1 and PAD6 in pre-motor PD.

### 3.3. Possible Roles for Citrullination/Deimination in the Gut-Brain Axis in PD 

Various infection-related pathways, including inflammatory and gut-brain axis pathways, were identified as citrullinated in the pre-motor PD brains in the current study. The neuro-microbiology of PD has received considerable interest [155]. Microglial activation and inflammatory responses linked to various pathogens, including parasitic, viral, and bacterial agents, have been reported [85]. Several infection and pathogen-linked KEGG pathways were identified in the current study associated with the brain citrulliomes of both control and pre-motor PD rats. Whether and how citrullination may play roles in escalating neuroinflammatory responses and downstream neurodegeneration in relation to such infection-associated pathways may be of considerable interest in future studies. The central roles of PADs in the CNS and their roles in various bacterial and viral infections, some of which are also related to neurological diseases [31,156,157,158], are of considerable interest in this context. This also includes long-term neurological outcomes in COVID-19 [86], which also has a gut-brain axis element, highlighting that further investigations may be of considerable interest in relation to downstream neurodegenerative pathways, including PD-related ones [87,159,160,161].

The gut, nasal and oral microbiota have received considerable attention in PD [162,163], but studies in relation to PADs and citrullination are still scarce. *Porphyromonas gingivalis* is strongly associated with citrullination in periodontitis [164] and has indeed been identified as one of various microbes in nervous tissues from several animal models of neurodegenerative diseases, including ALS, AD, and PD [165]. Importantly, the bacterial presence of *P. gingivalis*, which itself has citrullinating activity and may affect the host citrullinome [166], has been identified in the brain of both AD and PD based on 16S rRNA next-generation sequencing, assessing the early, intermediate, and late stage of the diseases. In previous studies, *P. gingivalis* has been reported in the hippocampus and cortex from control and PD donors [167] and identified to be mainly of oronasal origin. Interestingly, in AD, modification of the oral microbiome appears to be more prominent than in the gut, and AD studies have also focussed on *P. gingivalis* [168]. It was shown that oral gingivitis impairs gut permeability and mediates immune responses associated with neurodegeneration in LLRK2 PD mouse models of late-onset PD, and chronic periodontitis is a common type of peripheral inflammation associated with PD. It remains to be established whether *P. gingivalis*-induced dysbiosis plays a role in the pathophysiology of PD. Mice receiving oral *P. gingivalis* showed reduced dopaminergic neurones in the SN and the activation of microgial cells [169]. Interestingly, PD has been shown to alter the composition of the subgingival microbiome of periodontitis [170]. *P. gingivalis* has also been linked to rheumatoid arthritis (RA), one of the most extensively studied PAD-related pathologies to date, including in association with periodontal disease [171]. *P. gingivalis* has also been shown to accelerate atherosclerosis [172]. In RA, citrullination is identified in relation to intestinal permeability and microbial imbalance [173], and it must furthermore be considered that many other microbes may be able to cause citrullination of proteins in their hosts (both commensals and pathogens) due to bacterial PAD-homologues (ADI) [174,175], and this may contribute to citrullination mediated inflammatory responses exacerbating various pathologies. 

The composition of the faecal microbiota in the PD model treated with placebo has been shown to differ from that in the sham animals and to be correlated with increased plasma levels of inflammatory markers and neuroinflammation [176]. Further studies will be needed to characterise the possible presence and function of *P.gingivalis* in the PD model. In addition, it may be of interest in future studies to assess oral, nasal and gut citrullinomes in PD to establish which proteins in the brain can be modified by citrullination mediated by *P. gingivalis* or other types of infectious agents or commensals. The origin of PD onset has been debated, with some indication of origin in the gut, and PD is highlighted as a systemic inflammatory disease which is accompanied by bacterial inflammagens [177]. Hence, the question remains whether some of the suggested gut-brain axis involvement in PD may be linked to citrullination and while this has not been studied yet, various reports have highlighted potential roles for *P. gingivalis* in PD [178], including in PD with cognitive impairment [179].

### 3.4. Future Prospects for PAD Inhibitors in PD

Epigenetic mechanisms in PD, including post-translational modifications, are receiving increased interest, as highlighted in recent studies [4,5,180,181,182,183]. As PADs may be promising targets in PD therapeutics, roles for the different isozymes must be better understood for the development of therapeutics utilising pan-PAD or PAD isozyme-specific targeting. Pharmacological PAD inhibitors include the pan-PAD inhibitors Cl-amidine and BB-Cl-amidine, PAD2 inhibitor AMF30a, PAD3 inhibitor Cl4-amidine and PAD4 inhibitor GSK199 [184], some of which have been applied in various CNS in vivo and in vitro models [23,25,27,28,29,185]. Considering whether to choose pan-PAD versus isozyme-specific inhibitors may be important as PAD isozymes have different, and sometimes overlapping, preferences for target proteins; understanding the physiological and disease-related citrullinomes is, therefore, of importance. Future approaches for clinical PAD inhibitor treatment, both aimed at modulating total deimination/citrullination via pan-PAD inhibitors or a narrower range of deimination targets using PAD isozyme-specific inhibitors, still require further refinement and optimisation in experimental models. 

This is the first study to attempt a detailed mapping of citrullinome changes in different brain regions in PD, in this instance using a pre-motor toxin-induced PD rat model, and to identify brain-region-specific differences in PAD isozyme expression. The findings of this study align with those from other studies reporting modulation of PADs and citrullination in neurodegenerative diseases. It will be important, in continuation of this current study and previously published studies, to identify citrullination patterns in samples from human PD cohorts and link possible changes in circulatory PD signatures (plasma-EVs) to citrullination changes in brains. However, this will remain challenging as the citrullinome signatures from brains are only retrievable from post-mortem human samples. Comparisons with 6-OHDA in vitro human cell cultures, in addition to other PD animal models, including the A53T alpha-synuclein mouse model, which is linked to early-onset PD, and the A30P mouse model [186], may nevertheless provide some information to help move the field forwards. It will also be of interest in future studies to compare outcomes in the 6-OHDA-PD model to the LPS-PD model, which has recently highlighted the replication of crosstalk between local and systemic inflammatory response [107], which are inherent in PD pathogenesis and pathophysiology.

Based on recent studies and our current findings reported here, evidence is mounting for significant roles for PADs and citrullination in PD, including at early stages of the disease. By increasing our understanding of PAD-mediated brain-region-specific changes in disease progression, we will gain a better picture of the spatio-temporal roles of this post-translational modification in PD and its therapeutic potential. 

## 4. Materials and Methods

### 4.1. Pre-Motor PD Rat Model

This study employed the 6-hydroxydopamine (6-OHDA) rat model to induce pre-motor Parkinson’s disease (PD) in male Sprague-Dawley rats (200–250 g), adhering to ethical guidelines and regulatory approvals, including clearance from the Bloomsbury ethical committee and the Home Office as per the Animal Scientific Procedures Act 1986 (PPL PP3144142).

**Figure 9:**
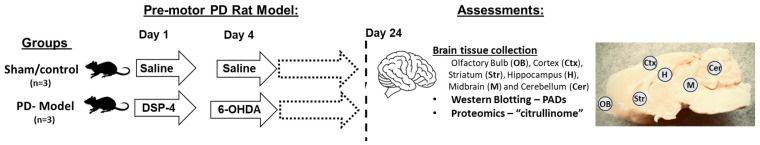
Experimental setup. Three animals per group were used for sham/control and the pre-motor PD model, respectively. Brains were extracted at the end of the treatment, and the six brain regions under study were excised and analysed for PADs by Western blotting and for brain-region-specific citrullinomes by proteomics analysis (LC-MS/MS).

The model, displaying non-motor symptoms without motor dysfunction, has been previously described in detail [4,187]. In brief, rats were randomly allocated to two experimental groups: Sham-treated (control) and toxin-treated model (PD); *n* = 3 animals per group were used for this study. The pre-motor PD model was established by intraperitoneal administration of either N-(2-chloroethyl)-N-ethyl-2-bromobenzylamine (DSP-4—25 mg/kg for the PD model) or sterile saline (for sham/controls) followed by bilateral striatal injections of 6-OHDA (15 µg per striatum—coordinates from Bregma: AP +1.0 mm, ML +3.0 mm, DV −6.5 mm—PD model) or saline (sham) containing 0.9% of ascorbic acid under general anaesthesia. Rats were maintained for 3 weeks after surgery and were then perfused. Brains were removed, and the different brain regions of interest (cortex, hippocampus, midbrain, striatum, cerebellum and olfactory bulb) were dissected, snap-frozen in liquid nitrogen, and stored at −80 °C for subsequent protein analysis. The experimental setup is summarised in Figure 9.

### 4.2. Protein Isolation from Brain Tissue 

Proteins were extracted from brain tissue from the different brain regions of PD-induced and control animals (*n* = 3 per group) according to previously described methods [40]. The six different brain regions under study (cortex, hippocampus, midbrain, striatum, cerebellum and olfactory bulb) were individually homogenised in RIPA+ buffer (Sigma-Aldrich, Gillingham, UK, containing 10% protease inhibitor cocktail, Sigma-Aldrich) in 2 mL Eppendorf tubes on ice using a Mini Handheld Homogeniser (Kimble, DWK Life Sciences, Lutterworth, UK). The homogenates were then gently pressed through a 23 G needle into fresh Eppendorf tubes on ice, followed by gently pipetting up and down to eliminate any tissue clots. For each 100 mg of tissue, 500 µL of RIPA+ buffer was used. The brain tissue homogenates were incubated for 1.5 h at 4 °C on a rolling platform, pipetting up and down at regular intervals. For protein isolation, the homogenates were then centrifuged at 16,000× *g* for 30 min at 4 °C, collecting the protein-containing supernatant, which was aliquoted and immediately frozen at −80 °C until further use. 

### 4.3. Isolation of Citrullinated/Deiminated Proteins from Brain Tissue

To identify the brain-region-specific citrullinomes, immunoprecipitation was carried out to isolate citrullinated/deiminated proteins from the different brain regions’ protein isolates using the F95 pan-citrulline antibody (MABN328, Merck, Watford, UK) [188]. For a representative citrullinome of each brain region (cortex, hippocampus, striatum, midbrain, cerebellum and olfactory bulb), protein extracts from 3 brains per experimental group (PD versus sham) were pooled (3 × 20 µL). Immunoprecipitation was carried out using the Catch and Release^®^ v2.0 Immunoprecipitation Kit (17-500M, Merck) together with the F95 pan-citrulline antibody and the affinity ligand provided with the kit, according to the manufacturer’s instructions (Merck). F95 enrichment was carried out overnight, incubating the mini-IP columns at 4 °C on a rotating platform. Thereafter, the citrullinated F95 bound proteins were eluted with the elution buffer provided with the kit, according to the manufacturer’s instructions (Merck), assessed by SDS-PAGE and silver staining (Silver Stain Plus Kit, BioRad, Watford, UK) for protein yield, and by LC-MS/MS analysis for the identification of individual citrullinated protein hits.

### 4.4. Liquid Chromatography with Tandem Mass Spectrometry (LC-MS/MS)

For LC-MS/MS analysis, the F95-enriched eluates from each brain region were run 0.5 cm into a 12% TGX gel (BioRad) and thereafter cut out as one band each, respectively, followed by in-gel digestion (Cambridge Proteomics, Cambridge, UK), according to previously described methods [4,40]. In brief, automated LC-MS/MS analysis was carried out using a Dionex Ultimate 3000 RSLC nanoUPLC (Thermo Fisher Scientific Inc., Waltham, MA, USA) system in conjunction with a QExactive Orbitrap mass spectrometer (Thermo Fisher Scientific Inc., Waltham, MA, USA). Peptide separation was carried out using reverse-phase chromatography and a Thermo Scientific reverse-phase nano Easy-spray column (Thermo Fisher Scientific Inc.). The LC eluent was sprayed into the mass spectrometer using an Easy-Spray source (Thermo Fisher Scientific Inc.). The m/z values of all eluting ions were measured in an Orbitrap mass analyser, and data-dependent scans (selecting the top 20) were employed for automatic isolation and generation of fragment ions using the HCD collision cell, measured using the Orbitrap analyser (Thermo Fisher Scientific Inc.). Both singly charged ions and ions with unassigned charge states were excluded from selection for MS/MS. A dynamic exclusion window of 20 s was also applied. Data were processed post-run using Protein Discoverer (version 2.1., Thermo Fisher Scientific Inc.), converted to mgf files and submitted to Mascot (Mascot search algorithm; Matrix Science, London, UK). A search for hits was carried out against the UniProt *Rattus_norvegicus*_20181203 (31,558 sequences; 17,280,660 residues) database with peptide and fragment mass tolerances, respectively, set at 20 ppm and 0.1 Da. The threshold value for significance was set at *p* < 0.05, and the peptide cut-off score was set at 35. 

### 4.5. Protein–Protein Interaction Network Analysis

To identify protein–protein interaction networks for rat-specific citrullinated/deiminated proteins hits from the six different brain regions (brain-region-specific citrullinomes), STRING analysis (Search Tool for the Retrieval of Interacting Genes/Proteins; https://string-db.org/) was used (accessed on 18 and 19 March 2024). The following functions were applied in STRING: “search multiple proteins”, the species database chosen was “*Rattus norvegicus*”, and basic settings and medium confidence were applied. Colour lines between the nodes indicate the following evidence-based interactions for network edges: “known interactions” (based on curated databases, experimentally determined), as well as “predicted interactions” (based on gene neighbourhood, gene fusion and gene co-occurrence, or via text mining, co-expression, or protein homology). Data for the pathway analysis of the protein networks were exported as STRING network images and as Excel files for KEGG and GO pathways and compared between brain regions from PD and sham rats, respectively.

### 4.6. Western Blotting

For Western blotting, a 100 µL aliquot of protein extract per sample was diluted with 100 µL 2× reducing Laemmli sample buffer (BioRad; containing 5% β-mercaptoethanol, Sigma-Aldrich, Gillingham, UK) and boiled for 5 min at 100 °C. Samples were run by SDS-PAGE (4–20% TGX gels, BioRad, Watford, UK) at 165 V for 52 min, using a 5 µL aliquot per sample per lane. Proteins were transferred to nitrocellulose membranes using semi-dry transfer (1h at 15V), assessing even protein transfer by PonceauS red stain (Sigma-Aldrich) before blocking in 5% bovine serum albumin (BSA, Sigma-Aldrich) in TBS-T for 1 h at room temperature (RT). For the detection of PAD isozymes, the membranes were incubated in primary antibodies overnight at 4 °C on a shaking platform as follows: PAD isozyme-specific antibodies used were anti-human PAD1 (ab181762, Abcam, Cambridge, UK), PAD2 (ab50257), PAD3 (ab50246), PAD4 (ab50247) and PAD6 (PA5-72059, Thermo Fisher Scientific, Hemel Hempstead, UK). All primary antibodies were used at 1/1000 dilution in TBS-T. Washing was carried out with TBS-T (3 × 10 min). Secondary antibody incubation was completed for 1 h at RT (using HRP-labelled anti-rabbit IgG; BioRad, diluted 1/3000 in TBS-T). Following washing (5 × 10 min in TBS-T), visualisation was carried out using ECL (Amersham Biosciences, Buckinghamshire, UK) and the UVP BioDoc-ITTM System (Thermo Fisher Scientific, Dartford, UK). All blots were re-probed with HRP-conjugated anti-β-actin antibody (ab20272, Abcam, 1/5000 in TBS-T), developed and imaged. For quantitative analysis of PAD isozymes, regions for protein bands in the expected size range of 70–75 kDa for PAD1–4 and 50–60 kDa for PAD6 were normalised against β-actin-positive bands following densitometry analysis using ImageJ (version 1.54g).

### 4.7. Statistical Analysis

For comparison between datasets from PD versus control brains, GraphPad Prism version 10 was used. T-tests were used to determine significance between groups for densitometry readings from Western blotting analysis, showing mean and standard deviation (*n* = 3 per experimental group). Statistical significance was regarded as *p* < 0.05. STRING analysis was carried out with medium confidence in STRING (https://string-db.org/, accessed on 18–19 March 2024).

## 5. Conclusions

This is the first study attempting a detailed mapping of citrullinome changes in different brain regions in PD using a pre-motor toxin-induced PD rat model. The findings of the study highlight some brain-region-specific differences in citrullinomes of cortex, hippocampus, cerebellum, striatum, midbrain and olfactory bulb of control versus pre-motor PD rat brains. We report both overlapping, control/sham and PD-specific KEGG and gene ontology (GO) pathways associated with the brain-region-specific citrullinomes relating to metabolic, immune, cell signalling and neurodegenerative disease-related pathways. Our findings identify roles for PAD-mediated citrullination in physiological and pathobiological processes, including in the early stages of PD, highlighting their potential for future therapeutic avenues. 

## Figures and Tables

**Table 1:** KEGG (Kyoto Encyclopedia of Genes and Genomes) pathways associated with the brain-region-specific citrullinomes. Protein–protein interaction networks were built based on citrullinated protein hits names per brain region (cortex CTX, hippocampus HIP, striatum STR, olfactory bulb OB, midbrain MID, cerebellum CER), in sham/control (ctr) and pre-motor PD (PD) brains. KEGG pathways were identified in STRING. All KEGG pathways identified are listed below, and a tick (V) indicates the KEGG pathway associated with each brain-region-specific citrullinome. The columns for the PD brain regions are highlighted in grey.

KEGG Pathway	Ctr CTX	PD CTX	Ctrl HIP	PD HIP	Ctr STR	PD STR	Ctr OB	PD OB	Ctr MB	PD MB	Ctr CER	PD CER
Oxidative phosphorylation	V	V	V	V	V	V	V		V			V
Synaptic vesicle cycle	V	V	V	V					V	V		V
Ribosome	V	V	V	V		V	V		V		V	
Parkinson’s disease	V	V	V	V	V	V	V		V	V	V	V
Retrograde endocannabinoid signalling	V	V	V	V			V		V			
Huntington disease	V	V	V	V	V	V	V		V	V	V	V
Prion disease	V	V	V	V	V	V	V		V	V	V	V
Gap junction	V	V	V	V			V		V	V		V
Non-alcoholic fatty liver disease	V	V	V	V	V		V		V			
Thermogenesis	V	V	V	V	V	V	V		V	V		V
Alzheimer’s disease	V	V	V	V	V	V	V		V	V		V
GABAergic synapse	V	V	V	V					V			
Amyotrophic lateral sclerosis	V	V	V	V	V		V		V	V	V	V
Endocrine and other factor-regulated calcium reabsorption	V			V								
Glutamatergic synapse	V	V	V	V			V		V			V
Phagosome	V	V	V	V			V		V	V		V
Cardiac muscle contraction	V	V	V	V								
Biosynthesis of amino acids	V	V	V	V			V		V		V	
Carbon metabolism	V	V	V	V					V			
Oestrogen signalling pathway	V	V	V	V		V	V	V	V	V	V	V
Necroptosis	V	V	V	V					V			
Endocytosis	V	V	V	V					V	V		V
Metabolic pathways	V	V	V	V			V		V			
Cocaine addiction	V	V										
Insulin secretion	V	V	V	V								
Apoptosis	V	V	V	V						V		V
cGMP-PKG signalling pathway	V	V		V								
Alcoholism	V		V	V								
Pyruvate metabolism	V	V	V	V					V		V	
Inositol phosphate metabolism		V										
Phosphatidylinositol signalling system		V		V								
Progesterone-mediated oocyte maturation		V										
Axon guidance		V										
Oocyte meiosis		V		V								
Cholinergic synapse	V	V	V	V								
Spinocerebellar ataxia		V										
Phospholipase D signalling pathway	V											
Rap1 signalling pathway		V										
2-Oxocarboxylic acid metabolism		V	V									
Nitrogen metabolism		V	V						V	V		
Pentose phosphate pathway		V	V									
Fructose and mannose metabolism		V	V	V					V			
Bacterial invasion of epithelial cells	V	V	V	V						V		
Leukocyte transendothelial migration		V	V									
Morphine addiction	V	V	V	V								
Regulation of actin cytoskeleton		V	V	V								
Citrate cycle (TCA cycle)	V	V	V	V					V			
Vasopressin-regulated water reabsorption		V	V	V								
Arginine biosynthesis		V	V	V					V			
Glycolysis/Gluconeogenesis	V	V	V	V			V		V		V	
Central carbon metabolism in cancer	V	V	V	V					V			
SNARE interactions in vesicular transport		V	V	V								
Alanine, aspartate and glutamate metabolism		V	V	V					V			
Cysteine and methionine metabolism		V	V	V					V			
HIF-1 signalling pathway	V	V	V	V					V			
Legionellosis			V	V					V		V	V
Salmonella infection	V	V	V	V								
Oxytocin signalling pathway	V	V	V	V								
Tight junction		V	V	V								
mTOR signalling pathway			V									
Viral carcinogenesis		V	V	V					V		V	
Collecting duct acid secretion			V									
Glyoxylate and dicarboxylate metabolism			V	V					V			
Butanoate metabolism			V									
Dopaminergic synapse	V	V	V	V								
Calcium signalling pathway	V	V	V	V								
Ferroptosis			V	V								
Systemic lupus erythematosus			V	V								
Long-term depression	V			V								
Hippo signalling pathway				V								
*Staphylococcus aureus* infection						V	V	V		V	V	
Gastric acid secretion		V	V				V				V	
Arrhythmogenic right ventricular cardiomyopathy							V	V		V		
Influenza A							V					
Glucagon signalling pathway	V	V	V	V			V		V		V	
GnRH secretion	V											
Type II diabetes mellitus	V	V	V	V								
Apelin signalling pathway	V											
Proteoglycans in cancer	V	V	V									
NOD-like receptor signalling pathway		V										
Platelet activation		V										
Cellular senescence	V	V										
Yersinia infection		V										
Human immunodeficiency virus 1 infection	V	V	V	V								
Propanoate metabolism			V	V								
Serotonergic synapse	V		V	V								
Beta-Alanine metabolism			V									
Thyroid hormone signalling pathway				V								
Fc gamma R-mediated phagocytosis				V								
Choline metabolism in cancer				V								
Spliceosome											V	
Antigen processing and presentation									V		V	
Protein processing in endoplasmic reticulum									V			
Human cytomegalovirus infection		V										

**Table 2:** Summary of PAD isozyme (PAD1, 2, 3, 4 and 6) detection, as assessed by Western blotting in the sham/control (CTR—white) and pre-motor PD (PD—grey) cortex, hippocampus, cerebellum, midbrain, olfactory bulb and striatum. Scoring, following normalisation with beta-actin, is shown as + low positive, ++ medium positive and +++ high positive. Brackets () indicate that the full score was not reached; statistically significant differences (*p* < 0.05) are indicated by a red star (*); Western blots are provided in Figures S1–S3.

Brain Region	Exp Group	PAD1	PAD2	PAD3	PAD4	PAD6
Cortex	CTR	(+)	++	+	+++	++
Cortex	PD	(+)	+++ *	++ *	+++	+++
Hippocampus	CTR	(+)	+	++	++	++
Hippocampus	PD	(+)	(+)	+(+)	+(+)	++(+)
Cerebellum	CTR	+	++	+	+	+
Cerebellum	PD	+	++	++ *	+(+) *	+(+) *
Midbrain	CTR	(+)	++	+(+)	+(+)	++
Midbrain	PD	+	++	+(+)	+	++
Olfactory bulb	CTR	(+)	+	+	+(+)	+
Olfactory bulb	PD	+	+	+(+) *	++	++
Striatum	CTR	++	+++	++	+++	+
Striatum	PD	+ *	+*	(+) *	+ *	(+) *

## Data Availability

All data supporting the study are included in the article and Supplementary Materials.

## Supplementary Materials

The following supporting information can be downloaded at https://www.mdpi.com/article/10.3390/ijms252011168/s1.
