# Supplementary material for: Brain-Region-Specific Differences in Protein Citrullination/Deimination in a Pre-Motor Parkinson’s Disease Rat Model
Source: Int J Mol Sci. 2024 Oct 17;25(20):11168. doi: 10.3390/ijms252011168 (PMC11509057; doi:10.3390/ijms252011168)
Supplement: Supplementary file 1 [file ijms-25-11168-s001.zip › ijms-3237227-supplementary Figures S1-S3.pdf]

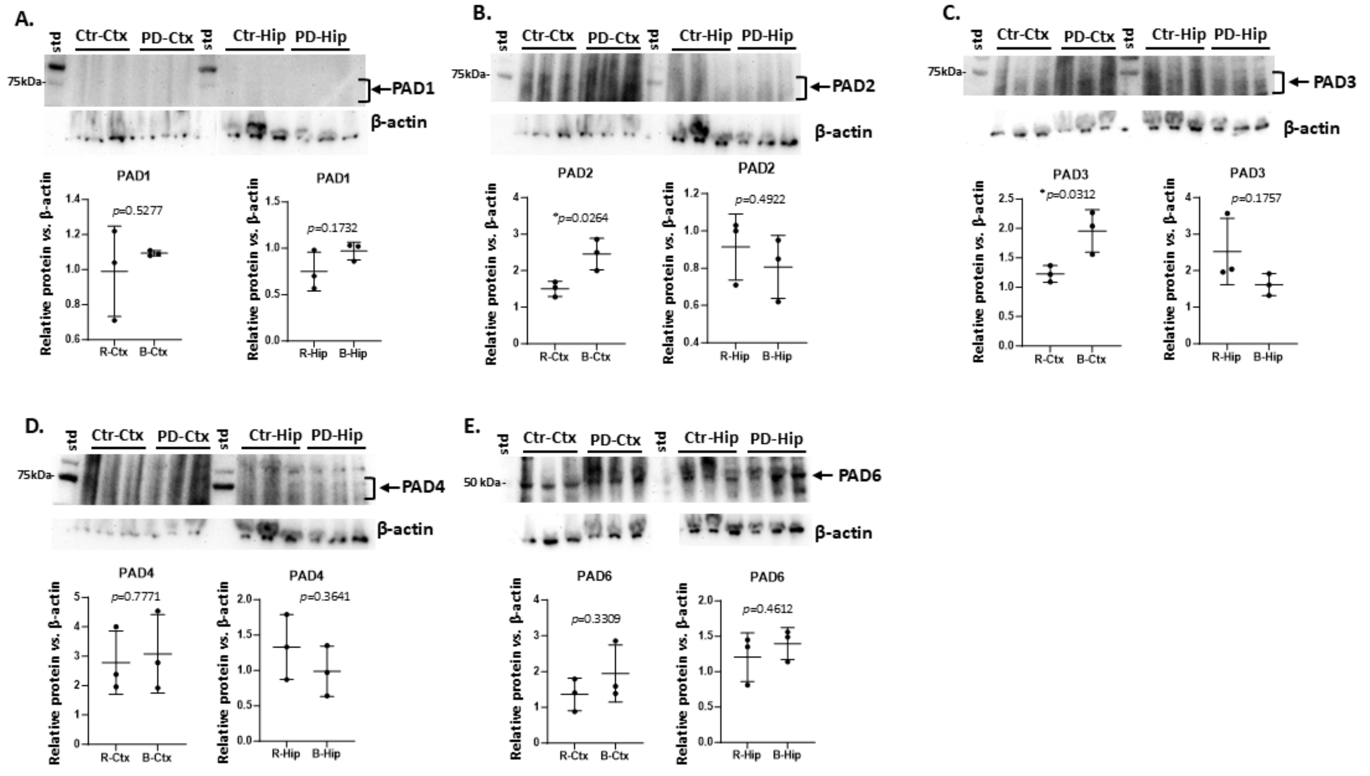

Figure S1. PAD isozyme protein detection in cortex (Ctx) and hippocampus (Hip) of sham/control (Ctr) and pre-motor PD (PD) rats. **A)** PAD1; **B)** PAD2; **C)** PAD3; **D)** PAD4; **E)** PAD6. Protein levels were assessed in n = 3 brains per group and normalised against beta-actin protein levels (fold-changes are shown); exact *p*-values are shown with significant differences indicated with a star *($p<0.05$); mean and standard deviation are presented. Arrows (and brackets) indicate the area of the blot assessed for the predicted PAD protein bands (at 70-75 kDa for PAD1-4 and 50-60 kDa for PAD6); note low detection of PAD1 in both cortex and hippocampus.

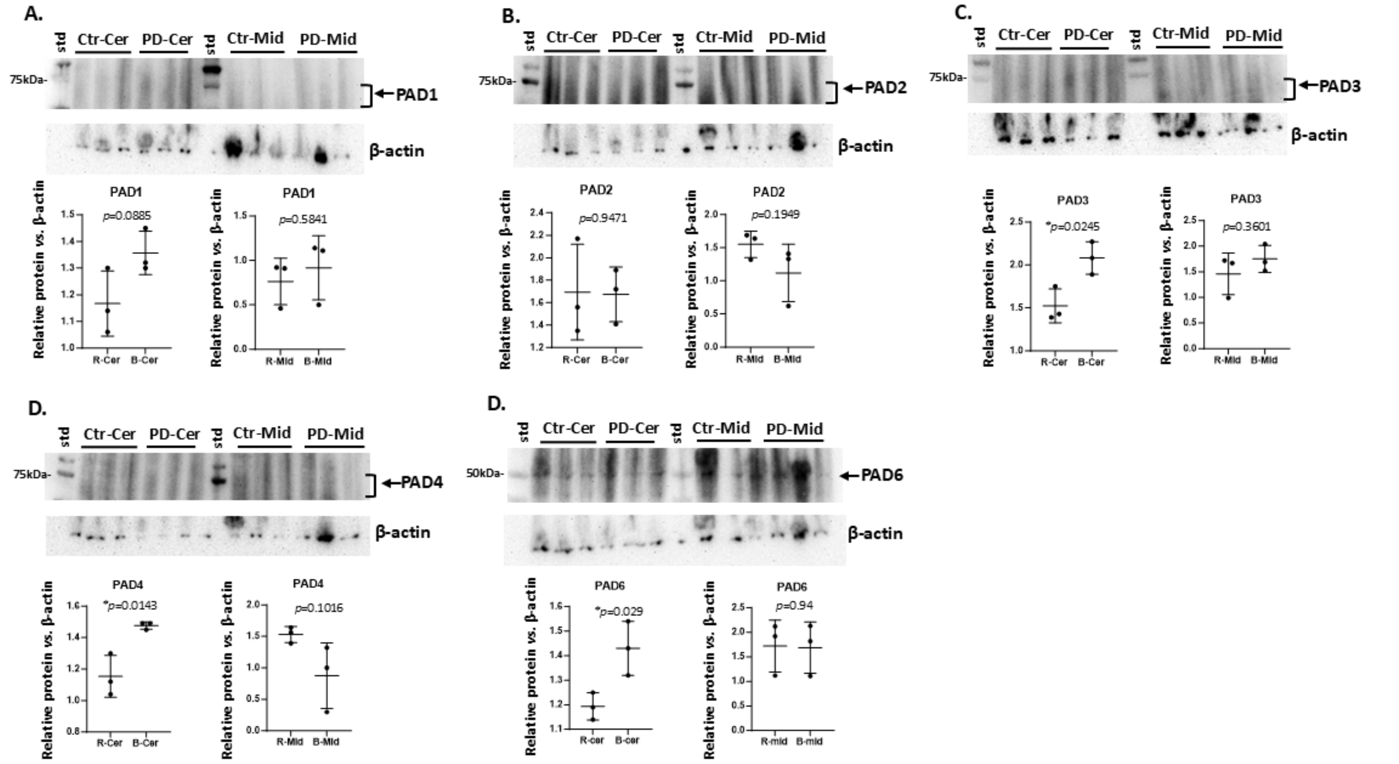

Figure S2. PAD isozyme detection in cerebellum (Cer) and midbrain (Mid) of sham/control (Ctr) and pre-motor PD (PD) rats. **A)** PAD1; **B)** PAD2; **C)** PAD3; **D)** PAD4; **E)** PAD6. Protein levels were assessed in n = 3 brains per group and normalised against beta-actin protein levels (fold-changes are shown); exact *p*-values are shown with significant differences indicated with a star *(p<0.05); mean and standard deviation are presented. Arrows (and brackets) indicate the area of the blot assessed for the predicted PAD protein bands (at 70-75 kDa for PAD1-4 and 50-60 kDa for PAD6).

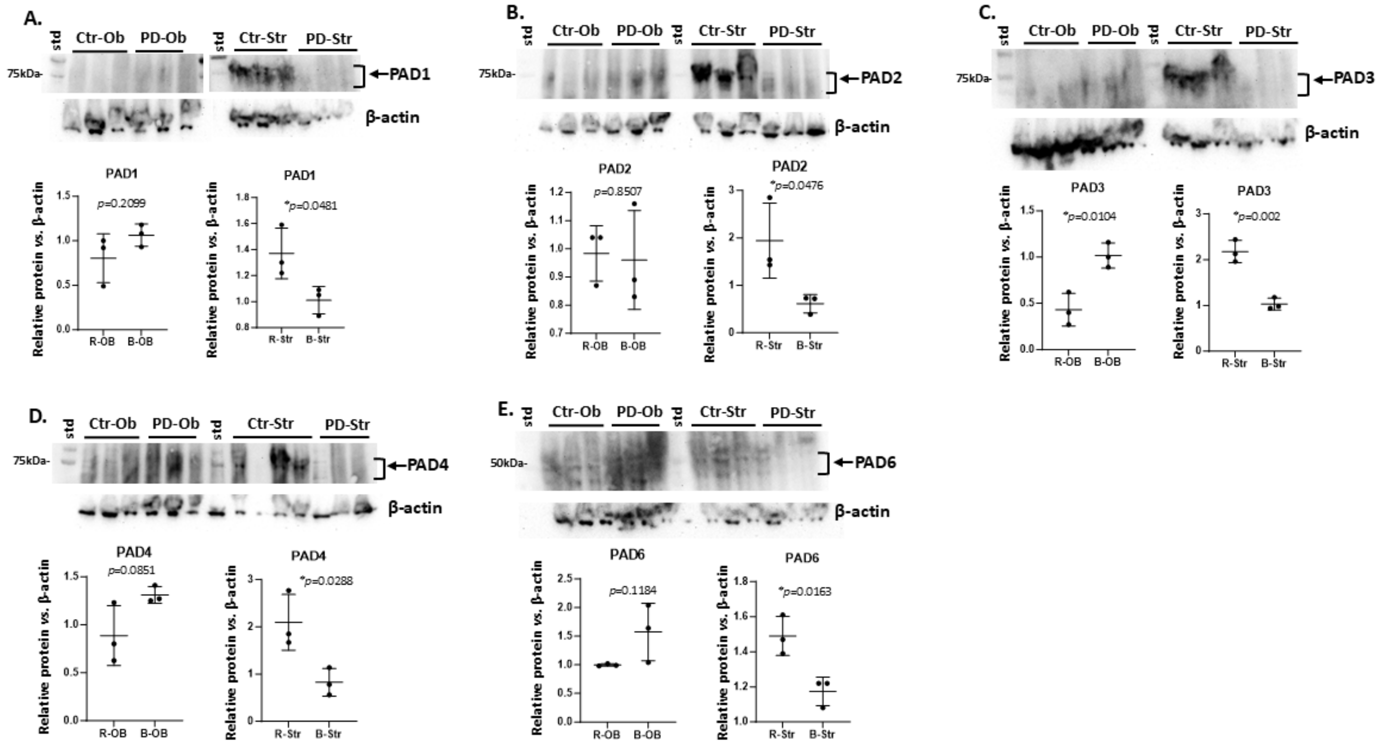

Figure S3. PAD isozyme protein detection in olfactory bulb (Ob) and striatum (Str) of sham/control (Ctr) and pre-motor PD (PD) rats. **A)** PAD1; **B)** PAD2; **C)** PAD3; **D)** PAD4; **E)** PAD6. Protein levels were assessed in n = 3 brains per group and normalised against beta-actin protein levels (fold-changes are shown); exact *p*-values are shown with significant differences indicated with a star *(p<0.05); mean and standard deviation are presented. Arrows (and brackets) indicate the area of the blot assessed for the predicted PAD protein bands (at 70-75 kDa for PAD1-4 and 50-60 kDa for PAD6).
